# DTI-ALPS Is Associated with Temporolimbic Amyloid but Not Plasma p-Tau181 Across the Alzheimer’s Disease Continuum

**DOI:** 10.21203/rs.3.rs-9382890/v1

**Published:** 2026-05-06

**Authors:** Rasa Zafari, Amirhossein Kamroo, Tina Taherkhani, Mahsa Heidari-Foroozan, Fardin Nabizdeh, Mohammad Hadi Aarabi

**Affiliations:** Tehran University of Medical Sciences; Tehran University of Medical Sciences; Tehran University of Medical Sciences; Shahid Beheshti University of Medical Sciences; Iran University of Medical Sciences; Université de Bordeaux, CNRS, CEA

**Keywords:** Alzheimer’s Disease, Mild Cognitive Impairment, Perivascular clearance, Amyloid, Glymphatic System, DTI-ALPS index

## Abstract

**Background:**

Alzheimer's disease (AD) is the most common cause of dementia, characterized by progressive aggregation of misfolded proteins. Accumulation of amyloid-beta (Aβ) and hyperphosphorylated tau (p-tau) is the hallmark pathology of AD. Diffusion tensor imaging along the perivascular spaces (DTI-ALPS) has been proposed as an MRI marker related to perivascular diffusivity patterns and has been used in studies of neurodegenerative disease.

**Aims:**

This study investigates the association between the association between DTI-ALPS indices, regional amyloid-PET burden, and plasma p-tau181 across the AD spectrum.

**Methods:**

Data from 410 individuals was enrolled from the Alzheimer's Disease Neuroimaging Initiative (ADNI) database. DTI-ALPS was used as an imaging proxy related to perivascular diffusivity patterns.

**Results:**

Mean DTI-ALPS declined progressively from healthy controls to AD. Moreover, females reflected higher DTI-ALPS indices compared with males. No significant associations were observed between perivascular diffusivity patterns and plasma concentration of p-tau 181 in patients with cognitive decline. In contrast, the global cortical amyloid-PET SUVR was associated with the mean [β = −0.163] and right DTI-ALPS indices in the MCI group [β = −0.202]. Moreover, we observed stronger associations between DTI-ALPS index and amyloid-PET SUVR in the temporal pole cortex [β = −0.199], entorhinal cortex [β = −0.224], and parahippocampal gyrus in patients with MCI [β = −0.281].

**Conclusions:**

Reduced DTI-ALPS indices were associated with increased temporolimbic amyloid deposition, particularly in individuals with MCI. These findings suggest DTI-ALPS-derived perivascular diffusivity as an imaging marker associated with amyloid burden in prodromal AD.

## Introduction

Alzheimer's disease (AD) is the most prevalent cause of dementia worldwide and it is a prominent challenge for healthcare providers, due to its considerable socioeconomic burden [1, 2]. The central driver for the development of AD is the aggregation of misfolded amyloid-beta (Aβ) plaques and hyperphosphorylated tau (p-tau) proteins [3–5]. Beyond their central role in AD pathophysiology, Aβ and p-tau are measurable hallmarks of disease pathology [6, 7]. Regarding the p-tau proteins, different p-tau isoforms of p-tau 231, p-tau 217, and p-tau 181 have been extensively investigated in the context of AD [8–10]. Specifically, p-tau 181 is a well-established indicator of neuronal damage in AD, which has revealed promising evaluation accuracy both in cerebrospinal fluid (CSF) sampling and positron emission tomography (PET) [11–14]. It is extensively reported that plasma p-tau181 represents downstream tau-related neurodegeneration and may reflect a systemic correlate of protein accumulation within the central nervous system (CNS) [15, 16]. While plasma p-tau181 reflects tau pathology and neurodegeneration, it remains unclear whether circulating tau levels are directly influenced by alterations in perivascular clearance mechanisms.

Beyond excessive protein aggregation, emerging evidence implies dysfunction of the neurovascular unit and brain barrier systems in the pathogenesis of AD [17]. It is shown that age-related alterations in the blood-brain barrier (BBB), perivascular spaces, and astrocytic aquaporin-4 (AQP4) polarization can result in impaired clearance of Aβ and p-tau in the brain tissue [18]. Such clearance failure is proposed to contribute to neurodegenerative processes [18].

As mentioned, recent studies have suggested that impairments in the clearance system of the brain can contribute to the excessive accumulation of p-tau deposits [19]. The glymphatic system is a pivotal clearance system in the brain, involved in the drainage of harmful waste, and plays a vital role in preserving the normal function of the brain [20, 21]. The function of the glymphatic system heavily relies on brain perivascular spaces (PVSs), consisting of periarterial spaces in arteries and perivenous spaces in veins [22]. Periarterial spaces are the entrance pathway for the influx of CSF into the brain parenchyma [23]. After the entry, AQP-4 channels of the astrocytes allow further fluid and material exchange between the CSF of the periarterial spaces and the interstitial fluid (ISF) [24]. Followed by the exchange, the efflux of the waste-containing CSF occurs through the perivenous spaces [25]. Interruptions in the function of this perivascular exchange system, particularly in aging, can exacerbate regional accumulation of misfolded proteins, particularly within vulnerable temporolimbic circuits [26]. Different approaches could be implemented for the clinical assessment of the glymphatic system, including infrared (IR) imaging [27], single-photon emission computed tomography (SPECT) [28], and contrast-enhanced or non-contrast magnetic resonance imaging (MRI) [29, 30]. Among the non-contrast MRI-based techniques, diffusion tensor imaging-based approaches (DTI) have been used to derive indices related to perivascular water diffusivity, which may reflect aspects of perivascular fluid dynamics [31].

DTI measures the diffusion of water molecules in the anatomical structures. The type of DTI used for the assessment of the fluid dynamics in the glymphatic system is called DTI along the perivascular spaces (DTI-ALPS) [32]. The DTI-ALPS measures water diffusion in three axes of subcortical fibers, association fibers, and projection fibers at the level of the lateral ventricle body [33]. Subcortical fibers as the X-axis, association fibers as the Y-axis, and projection fibers serve as the Z-axis [34]. Ultimately, the ratio of X-axis water diffusion to the average of Y/Z-axes water diffusion indicates the DTI-ALPS quantified index that has been proposed as an indirect imaging marker related to water diffusivity along perivascular spaces, and has been interpreted as a potential proxy of perivascular fluid dynamics [35].

Therefore, in this study, we aimed to examine whether DTI-ALPS indices are associated with regional amyloid deposition in temporolimbic structures and whether such alterations relate to circulating p-tau181 across the Alzheimer’s disease continuum. We hypothesized that the altered diffusivity in perivascular system would be associated with an exacerbated accumulation of Aβ in the brain and potentially elevated plasma levels of p-tau 181 among patients with cognitive impairment. This study provides a more in-depth insight into the neuronal pathophysiology and abnormal functionality of the glymphatic system in patients with cognitive impairments.

## Methods

### Subject Cohort and Data Acquisition

Data for 410 individuals, including cognitively normal controls and participants with varying degrees of cognitive impairment were obtained from the Alzheimer's Disease Neuroimaging Initiative (ADNI) database. Inclusion of the participants relied on the availability of complete diffusion-weighted imaging (DWI) sequences, T1-weighted anatomical scans, plasma biomarker data and amyloid-PET measurements. Datasets that showed significant motion artifact or structural anomalies, as determined by standardized ADNI quality control protocols, were excluded. Demographic covariates including age, sex, years of education, clinical scores comprised of MMSE and ADAS-11, APOE ε4 status, and biomarker levels were extracted.

### Magnetic Resonance Imaging Acquisition

All imaging data were acquired according to the standardized ADNI acquisition protocol across multiple sites. 3 Tesla MRI systems were used. The diffusion-weighted imaging protocol utilized multiple non-colinear gradient directions with a b-value of 1000 s/mm^2^, supplemented by multiple b0 reference images. High-resolution 3D T1-weighted anatomical images were obtained at 1.0 mm isotropic resolution. Amyloid-PET data were processed and quantified as standard uptake value ratio (SUVR) in accordance with ADNI pipelines.

### Diffusion Data Preprocessing and Tensor Modeling

Diffusion-weighted images were first visually inspected to ensure the absence of gross artifacts and motion-related distortions. Preprocessing was then performed using the FMRIB Software Library (FSL, version 6.0) and MRtrix3 [36, 37]. The diffusion data were denoised using the Marchenko-Pastur principal component analysis–based algorithm (dwidenoise) [38] and corrected for Gibbs ringing artifacts (mrdegibbs) [39]. Eddy-current and motion-induced distortions were corrected using FSL’s eddy, with outlier replacement and slice-to-volume motion correction enabled when appropriate.

Following preprocessing, diffusion n tensors were fitted voxel-wise using FSL’s dtifit to generate diffusion tensor–derived maps, including the three principal diffusivities (Dxx, Dyy, Dzz) corresponding to diffusivity along the x-, y-, and z-axes of the diffusion tensor, respectively. These maps were subsequently used for the calculation of the DTI-ALPS index [32]. Fractional anisotropy (FA) maps were also generated and used for spatial normalization to the JHU-ICBM 1 mm FA template using linear registration (flirt) [40]. All processing steps were carried out in the native diffusion space prior to registration to minimize interpolation effects.

### Quantification of Perivascular Fluid Dynamics

The function of the glymphatic system was calculated using the DTI-ALPS method. At the level of the centrum semiovale, the perivascular spaces accompanying the medullary veins run predominantly along the left–right (x) axis, while the projection fibers (within the superior corona radiata, SCR) are oriented in the superior-inferior (z) direction and the association fibers (within the superior longitudinal fasciculus, SLF) run in the anterior-posterior (y) direction. Because of this nearly orthogonal geometry, diffusion along the x-axis in this region primarily reflects water movement within perivascular channels, whereas diffusion along the y- and z-axes represents diffusion perpendicular to these spaces [41].

Four regions of interest (ROIs) were placed bilaterally in the left and right SCR and SLF using predefined templates in the JHU-ICBM FA space. The individual diffusion tensor components, Dxx, Dyy, and Dzz, representing diffusivity along the x, y, and z axes, respectively, were extracted from these ROIs. The DTI-ALPS index was computed separately for each hemisphere using the formula:

$$DTI-ALPS\;\text{index}=\frac{D_{x(\text{Projection ROI})}}{D_{y(\text{Projection ROI})}}+\frac{D_{x(\text{Association ROI})}}{D_{z(\text{Association ROI})}}$$

Dxx, Dyy, and Dzz correspond to the directional diffusivities within the projection (SCR) and association (SLF) fibers. A higher ALPS index indicates greater diffusivity along the perivascular-space axis, which has been interpreted as potentially reflecting more preserved perivascular fluid dynamics; however, it does not directly quantify glymphatic flow.

All diffusion metrics were calculated in the native diffusion space to minimize interpolation errors and then spatially normalized for group-level analysis. The per-subject ALPS index was exported to a tabular file for subsequent statistical analysis.

### Statistical Modeling Framework

All statistical analyses were performed using SPSS software (Version 20). The normality of variable distributions was evaluated using the Kolmogorov-Smirnov and Shapiro-Wilk tests. To evaluate the association between the DTI-ALPS index and core Alzheimer's disease biomarkers, multivariable linear regression models were used. The primary models assessed the relationship between the mean DTI-ALPS index (dependent variable) and plasma p-tau 181 levels, as well as global amyloid-PET SUVR, adjusted for the covariates of age, sex, and years of education. Secondary analyses examined hemispheric-specific indices and their relationship with amyloid-PET SUVR in the temporal pole cortex, inferior temporal cortex, middle temporal cortex, entorhinal cortex, and parahippocampal gyrus, due to their involvement in early-stage Alzheimer’s disease and their known vulnerability to amyloid accumulation. A p value < 0.05 was considered statistically significant. To account for multiple regional comparisons, false discovery rate (FDR) correction was applied using the Benjamini-Hochberg procedure across amyloid-PET regional analyses, with q < 0.05 considered statistically significant. In exploratory analyses, we also compared effect sizes across diagnostic groups to evaluate whether DTI-ALPS-amyloid associations were stage-specific (HC vs MCI vs AD), given the diagnostic focus of biomarker-based stratification.

## Results

### Demographic and clinical features of participants

**Figure 1:**
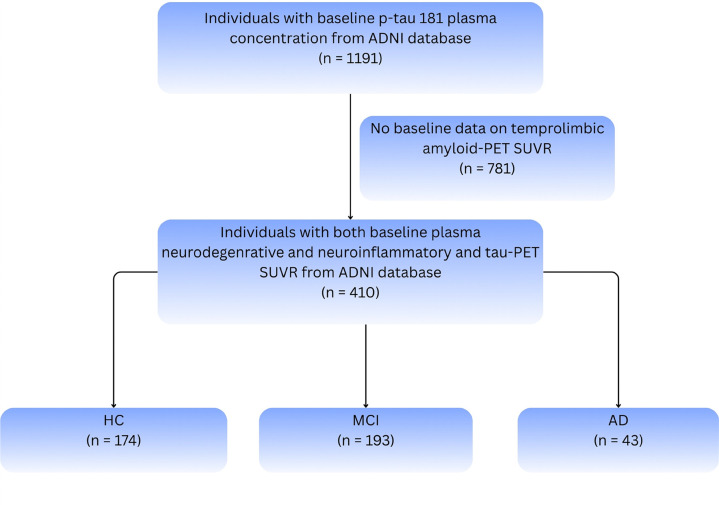
Flowchart of ADNI data applications. HC: Healthy controls, MCI: Mild cognitive impairment, AD: Alzheimer’s disease.

The baseline cohort data of 410 individuals, consisting of 217 males and 193 females, were used in this study (Fig. 1). The overall mean age was 71.91 ± 6.98, and the mean years of education was 16.21 ± 2.68 among all participants. In addition, the mean scores for the Mini-Mental State Examination (MMSE) and Alzheimer's Disease Assessment Scale (ADAS-11) were 27.96 ± 2.32 and 8.63 ± 6.07, respectively. Moreover, 42.19% of individuals reflected at least one APOE 4. We found a significant difference in the cognitive performance of participants in both the MMSE [F (2,407) = 224.356, P value < 0.001, η^2^ = 0.524] and the ADAS-11 questionnaires [F (2,407) = 196.801, P value < 0.001, η^2^ = 0.492].

**Figure 2:**
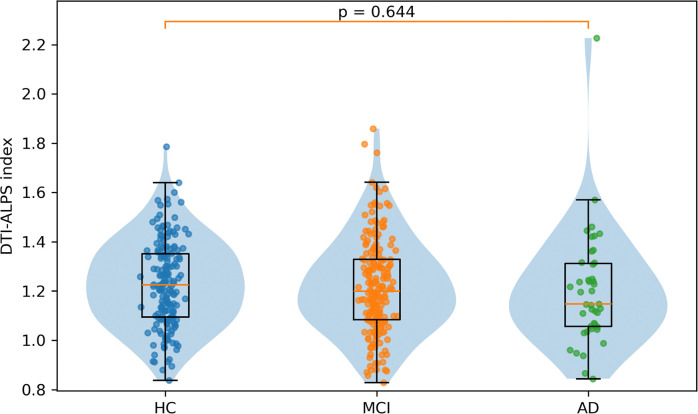
Baseline DTI-ALPS indices among participants. DTI-ALPS index: Diffusion Tensor Image Analysis along the Perivascular Space index, HC: Healthy controls, MCI: Mild cognitive impairment, AD: Alzheimer’s disease.

Nevertheless, the plasma level of p-tau 181 was significantly different among individuals with cognitive impairment and healthy controls [F (2,407) = 15.586, P value < 0.001, η^2^ = 0.071]. Considering imaging biomarkers, our analysis demonstrated considerable differences between groups in global cortical amyloid-PET SUVR [F (2,407) = 60.000, P value < 0.001, η^2^ = 0.228], as well as amyloid-PET SUVR in temporal pole cortex [F (2,407) = 15.797, P value < 0.001, η^2^ = 0.072], middle temporal cortex [F (2,407) = 37.188, P value < 0.001, η^2^ = 0.155], inferior temporal cortex [F (2,407) = 41.020, P value < 0.001, η^2^ = 0.168], and parahippocampal gyrus [F (2,407) = 25.319, P value < 0.001, η^2^ = 0.111]. Additionally, no significant differences were observed in the mean DTI-ALPS index among patients with cognitive impairment and healthy individuals [F (2,407) = 0.441, P value = 0.644, η^2^ = 0.002] (Fig. 2). Table 1 summarizes demographic characteristics of participants.

### Association of DTI-ALPS indices with cognitive performance and demographic characteristics

We conducted a linear regression model to investigate whether there is a significant association between DTI-ALPS indices and demographic features, as well as cognitive function among the participants. Unlike patients with AD [P value = 0.136], our analysis revealed a significant relationship between the DTI-ALPS index and the age of the healthy participants [standardized β = −0.314, P value < 0.001, 95% CI= −0.457 to −0.172, Adjusted R^2^= 0.099] and the MCI group [standardized β = −0.209, P value < 0.05, 95% CI= −0.349 to −0.070, Adjusted R^2^= 0.044]. However, no significant associations were shown between the DTI-ALPS index and the years of education among patients with AD [P value = 0.586] and healthy individuals [P value = 0.482]. Additionally, the DTI-ALPS index was not associated with the cognitive performance of participants with AD in MMSE [P value = 0.143] or ADAS-11 [P value = 0.166].

### Sex difference in DTI-ALPS indices

**Figure 3:**
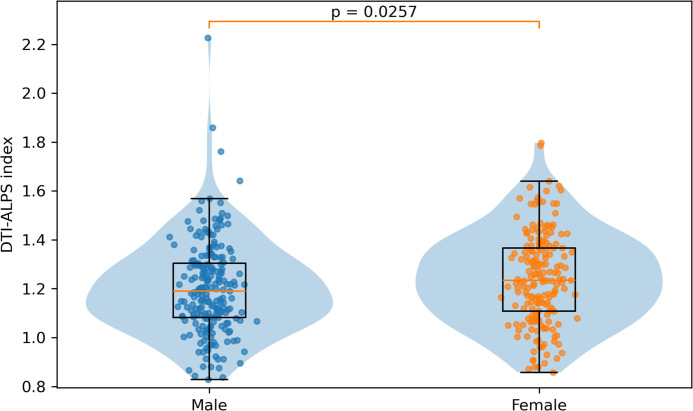
Sex differences in the function of the glymphatic system. Females demonstrated higher DTI-ALPS indices compared with males. DTI-ALPS index: Diffusion Tensor Image Analysis along the Perivascular Space index.

We found a significant association between the sex of healthy individuals and the mean DTI-ALPS index [standardized β = 0.264, P value < 0.001, 95% CI=0.119 to 0.409, Adjusted R^2^= 0.064]. Then, we conducted an independent t-test to compare the DTI-ALPS index among participants. Unlike patients with cognitive impairment, healthy participants reflected a significant difference in the DTI-ALPS index between the two sexes, with a higher glymphatic activity in females [t (173) = −3.594, P value < 0.001, η^2^ = 0.069]. Similar results on the significant difference of the glymphatic function were also observed among all participants [t (408) = −2.239, P value < 0.05, η^2^ = 0.012] (Fig. 3).

### Association of DTI-ALPS indices with plasma p-tau 181

**Figure 4:**
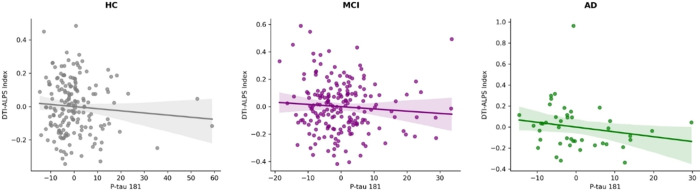
Association between baseline DTI-ALPS index and plasma p-tau 181. No significant associations were found between the baseline DTI-ALPS index and plasma p-tau 181 in any groups. DTI-ALPS index: Diffusion Tensor Image Analysis along the Perivascular Space index, HC: Healthy controls, MCI: Mild cognitive impairment, AD: Alzheimer’s disease.

To assess the association between DTI-ALPS indices and plasma levels of p-tau 181, we used a multivariable linear regression model adjusted for age, sex, APOE 4, and years of education. Our analysis reported a negative but insignificant association between the mean DTI-ALPS index and plasma concentrations of p-tau 181 among patients with AD [standardized β = −0.204, P value = 0.255, 95% CI= −0.561 to 0.153, Adjusted R^2^= 0.004]. Also, there were no significant associations between DTI-ALPS index and p-tau181 in patients with MCI or healthy participants (Fig. 4).

### Association of DTI-ALPS indices with Amyloid-PET

**Figure 5:**
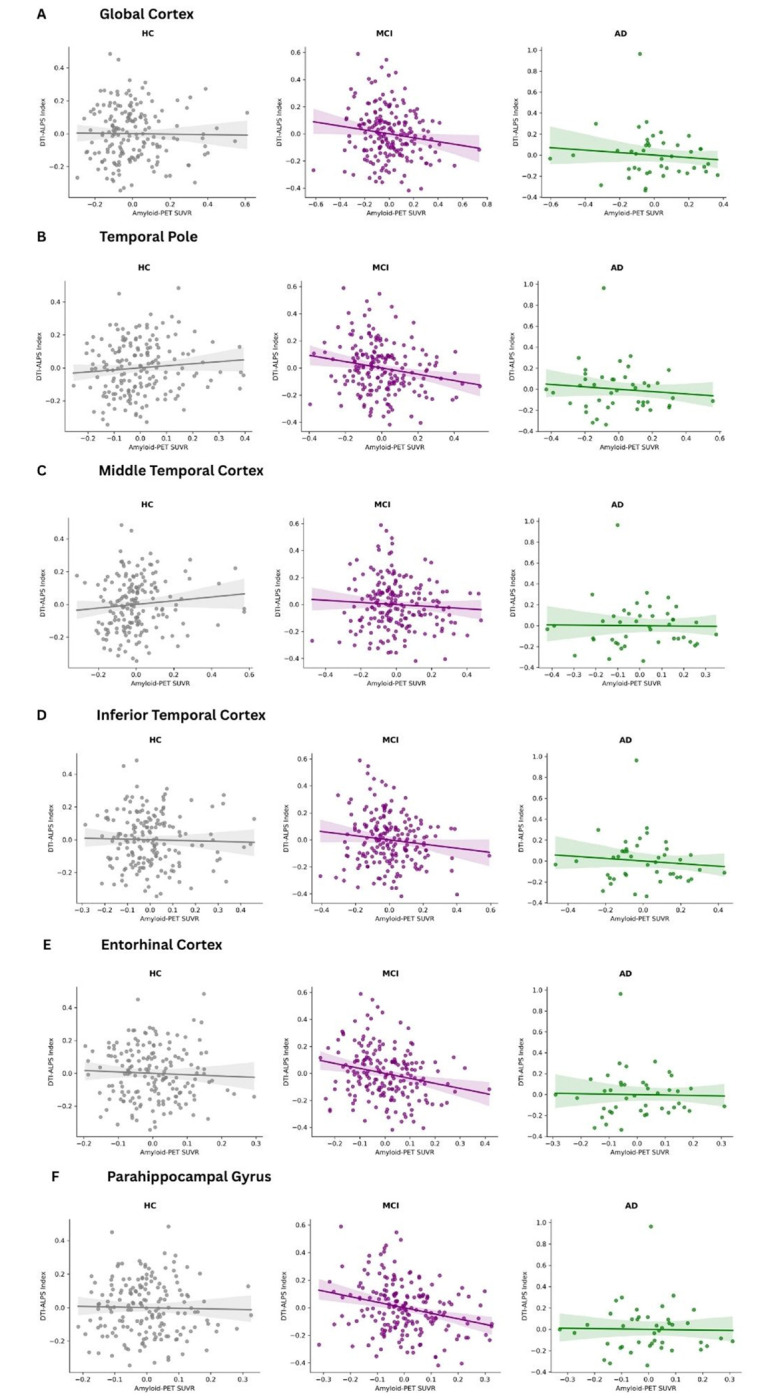
Association between DTI-ALPS index and regional amyloid-PET SUVR. Scatterplots show the relationship between baseline DTI-ALPS index and amyloid-PET SUVR in the global cortex (A), temporal pole (B), middle temporal cortex (C), inferior temporal cortex (D), entorhinal cortex (E), and parahippocampal gyrus (F), stratified by HC, MCI, and AD groups. Significant negative associations were observed primarily in the MCI group. SUVR: Standardized uptake value ratio, DTI-ALPS index: Diffusion Tensor Image Analysis along the Perivascular Space index, HC: Healthy controls, MCI: Mild cognitive impairment, AD: Alzheimer’s disease.

We observed the association between DTI-ALPS index and global cortical amyloid-PET SUVR, as well as amyloid SUVR in different brain regions, including the temporal cortex, the entorhinal cortex, and the parahippocampal gyrus. The amyloid-PET SUVR of the global cortex was negatively associated with the mean [standardized β = −0.109, P value = 0.047, 95% CI= −0.214 to −0.004, Adjusted R^2^= 0.073] and right DTI-ALPS indices among all participants [standardized β = −0.127, P value = 0.047, 95% CI= −0.233 to −0.022, Adjusted R^2^= 0.069]. However, after separating groups, only patients with MCI reflected significant associations, with global cortical SUVR linked to both the mean DTI-ALPS index [standardized β = −0.163, P = 0.047, 95% CI= −0.325 to −0.002, Adjusted R^2^=0.058] and the right DTI-ALPS index [standardized β = −0.202, P = 0.014, 95% CI= −0.364 to −0.041, Adjusted R^2^= 0.057] (Fig. 5A).

In the temporal pole cortex, we found no significant associations between DTI-ALPS index and amyloid uptake when considering all participants. In contrast, among patients with MCI, the temporal pole SUVR of amyloid was significantly associated with the mean DTI-ALPS index [standardized β = −0.199, P value < 0.05, 95% CI= −0.343 to −0.054, Adjusted R^2^= 0.074] (Fig. 5B), as well as the DTI-ALPS index in the right and left hemispheres [standardized β = − 0.196, P value < 0.05, 95% CI= −0.342 to −0.050, Adjusted R^2^= 0.062 in the right hemisphere VS standardized β = − 0.175, P value < 0.05, 95% CI= −0.320 to −0.029, Adjusted R^2^= 0.063 in the left hemisphere]. For the middle and inferior temporal cortices, no associations emerged in any group, except for a nominal association between the right DTI-ALPS index and amyloid-PET SUVR in the middle temporal cortex in patients with MCI [standardized β = −0.169, P value < 0.05, 95% CI= −0.332 to −0.007, Adjusted R^2^= 0.048], which did not survive FDR correction (Fig. 5C, D).

In the entorhinal cortex, lower glymphatic function was associated with more accumulated amyloid plaques when combining all participants [standardized β = −0.134, P value < 0.05, 95% CI= −0.229 to −0.039, Adjusted R^2^= 0.081]. In addition, unlike patients with AD, significant association was found between the mean DTI-ALPS index and amyloid-PET SUVR in the entorhinal cortex among patients with MCI [standardized β = −0.224, P value < 0.05, 95% CI= −0.363 to −0.084, Adjusted R^2^= 0.087] (Figure 5E). Also, a similar associational trend was shown between amyloid uptake in the entorhinal cortex and the glymphatic function in patients with MCI in the right [standardized β = −0.240, P value < 0.001, 95% CI= −0.380 to −0.100, Adjusted R^2^= 0.083], and left hemispheres [standardized β = −0.179, P value < 0.05, 95% CI= −0.320 to −0.038, Adjusted R^2^= 0.066].

In addition, among all participants, the mean DTI-ALPS index was negatively associated with amyloid-PET SUVR in the parahippocampal gyrus [standardized β = −0.151, P value < 0.05, 95% CI= −0.250 to −0.051, Adjusted R^2^= 0.084]. This trend was also true for glymphatic function in both the right and left hemispheres [standardized β = −0.154, P value < 0.05, 95% CI= −0.253 to −0.054, Adjusted R^2^= 0.022 in the right hemisphere Vs. standardized β = −0.129, P value < 0.05, 95% CI= −0.228 to −0.029, Adjusted R^2^= 0.070 in the left hemisphere]. Unlike the two other groups, impaired function of the glymphatic system was significantly associated with increased amyloid SUVR in the parahippocampal gyrus of individuals with MCI [standardized β = −0.281, P value < 0.001, 95% CI= −0.428 to −0.134, Adjusted R^2^= 0.106] (Figure 5F). This pattern was mirrored in both the right and left DTI-ALPS indices [standardized β = −0.290, P value < 0.001, 95% CI= −0.438 to −0.143, Adjusted R^2^= 0.099 in the right hemisphere Vs. standardized β = −0.236, P value < 0.05, 95% CI= −0.384 to −0.087, Adjusted R^2^= 0.083 in the left hemisphere] (Table 2). However, after FDR correction across all regional and hemispheric analyses in the MCI group, associations within the parahippocampal gyrus, entorhinal cortex, and temporal pole remained significant for mean, right, and left DTI-ALPS indices, while only the right DTI-ALPS association with global cortical amyloid survived correction.

## Discussion

To the best of our knowledge, the present study is the first investigation that observes the association between the DTI-ALPS index and plasma levels of p-tau 181, as well as the amyloid-PET findings among patients across the AD spectrum. We found that the DTI-ALPS index is negatively associated with the age of healthy participants. Additionally, a significant association was reported between the DTI-ALPS index and amyloid-PET SUVR in the entorhinal cortex and parahippocampal gyrus among all participants. It is also shown that unlike other temporal regions the amyloid-PET SUVR of the inferior temporal cortex was associated with the right DTI-ALPS index among all participants.

In addition, patients in the MCI group reflected a significant association between the glymphatic function in the right hemisphere, as assessed indirectly by the DTI-ALPS index, and amyloid-PET SUVR of the global cortex. In addition, the amyloid SUVR in the temporal pole, entorhinal cortex, and parahippocampal gyrus of patients with MCI was significantly associated with DTI-ALPS index. However, no associations were found between the perivascular diffusivity patterns and amyloid-PET findings in patients with AD and there were no significant associations between the DTI-ALPS index and the plasma level of p-tau181 in either group. The absence of associations between DTI-ALPS and plasma p-tau181 suggests that glymphatic impairment may relate more closely to regional cortical amyloid deposition than to circulating tau biomarkers.

The DTI-ALPS index was first proposed by Taoka et al. (2017) as an indirect imaging biomarker to assess the glymphatic system function [32]. The DTI-ALPS index is calculated by comparing water diffusivity along perivascular spaces with diffusivity in projection and association fibers at the level of the lateral ventricles [42]. Specifically, it is introduced as the ratio of diffusivity along the x-axis (parallel to perivascular spaces) to the average diffusivity along the y-and z-axes (perpendicular directions), reflecting the efficiency of fluid transport in perivascular pathways. The DTI-ALPS index, as an indicator of the perivascular diffusivity patterns, has been extensively utilized by previous studies to enhance understanding of neurodegenerative disorders. Recent studies have reported reduced DTI-ALPS indices in patients with AD and MCI, which have been interpreted as reflecting deficits in perivascular diffusivity patterns [43–47]. A recent meta-analysis on DTI-ALPS index in Parkinson’s disease (PD) and AD has reported not only significant changes in the DTI-ALPS index in either PD and AD in comparison to healthy controls, but also has reported an acceptable accuracy for the DTI-ALPS index in distinguishing patients with AD from those suffering from PD [48]. In contrast, our findings did not reflect a significant difference in the DTI-ALPS index between participants with AD spectrum and the control group. This result may be attributed to factors such as the small sample size, especially in the AD group, or heterogeneous disease stages.

We reported a negative association between the DTI-ALPS index and the age of participants. This finding aligns with the results of previous studies, reporting a considerable correlation between age and the function of the glymphatic system [49–51]. These findings suggest an impaired function for the perivascular diffusivity patterns in older individuals. Additionally, we demonstrated a significant difference in the DTI-ALPS index between the two sexes. The evidence also extensively reports higher amounts of the DTI-ALPS index in females than in males, both in healthy participants and those with various neurodegenerative disorders [48, 52–54]. This difference between the two sexes can be due to multiple reasons. It is shown that female sexual hormones, like estrogen and progesterone, increase the expression of AQP-4 channels and thus enhance the clearance throughout the glymphatic system [55]. Moreover, females have higher resting cerebral blood flow and their better arterial pulsatility can lead to increased DTI-ALPS index compared to males [56, 57].

P-tau 181, p-tau 217, and p-tau 231 are among the most commonly assessed biomarkers in studies investigating AD [58]. While it is shown that p-tau 217 and p-tau 231 increase in the early stages of AD continuum, compared to the plasma and CSF levels of p-tau 181, recent studies have suggested a similar diagnostic reliability for these biomarkers among patients with AD spectrum [59]. Moreover, p-tau 181 is shown to be a more accurate biomarker in the observation of the stage and severity of cognitive decline among patients with AD continuum [16]. Based on previous studies, p-tau 181 CSF levels and related PET findings have become strong biomarkers for the early diagnosis of AD; however, the invasive and high-cost evaluation process restricts their availability, particularly in developing countries [60]. Thus, finding an available imaging technique associated with established AD biomarkers can promise a more cost-effective and non-invasive method in monitoring disease progression among patients with cognitive decline.

However, our study revealed no considerable associations between the DTI-ALPS index and the plasma concentration of p-tau 181 among patients with AD spectrum. This finding is in line with previous studies, reporting no correlations between the DTI-ALPS index and plasma or CSF levels of p-tau 181 across different neurodegenerative diseases, such as PD and AD [61, 62]. The stronger associations observed in MCI may reflect that impairments of perivascular clearance exhibit more measurable influence during the prodromal stage of AD. In other words, widespread neurodegeneration, vascular remodeling, and structural tissue loss during advanced stages of the AD continuum may mask the association between fluid transport dysfunction and protein accumulation reported in early stages. This pattern aligns with models proposing that barrier and clearance dysfunction precede overt neuronal loss [63, 64]. Although, a recent mouse model of AD has demonstrated that impairment of AQP4 leads to increased p-tau 181 accumulation in the CSF [65]. Some studies have also revealed negative correlations between t-tau and p-tau181 CSF levels and DTI-ALPS index among patients with AD and MCI [66]. Such inconsistent findings highlight the need for more longitudinal studies with larger sample sizes to provide more sufficient evidence on the role of the glymphatic system in the clearance of tau tangles and to assess the validity of DTI-ALPS as a non-invasive imaging method in the assessment of disease progression among patients with AD continuum.

We chose the temporal pole cortex, inferior temporal cortex, middle temporal cortex, entorhinal cortex, and parahippocampal gyrus as our ROIs in amyloid-PET SUVR measurement. The temporal lobe is among the key areas of the brain tissue involved in AD and its damage can lead to considerable cognitive impairment among patients with AD spectrum [67]. Among the temporal lobe subregions, the temporal pole cortex, inferior temporal cortex, and middle temporal cortex are constantly reported to exhibit significant synaptic loss and cortical thinning, contributing to apathy in patients with cognitive impairment [68–70]. In addition, the parahippocampal gyrus and entorhinal cortex are among the brain regions affected in early stages of AD, causing deficits in recent memory and spatial navigation [71]. The predominance of associations within temporolimbic structures may reflect the vulnerability of these regions to impaired perivascular clearance. Therefore, impaired perivascular exchange in aging may preferentially facilitate protein accumulation in these anatomically vulnerable circuits. Moreover, these findings suggest that assessing the relationship between amyloid-PET burden in these ROIs and the DTI-ALPS index may offer deeper insights into how perivascular diffusivity imapirment contributes to waste accumulation within temporolimbic circuits across the AD spectrum.

**Figure 6:**
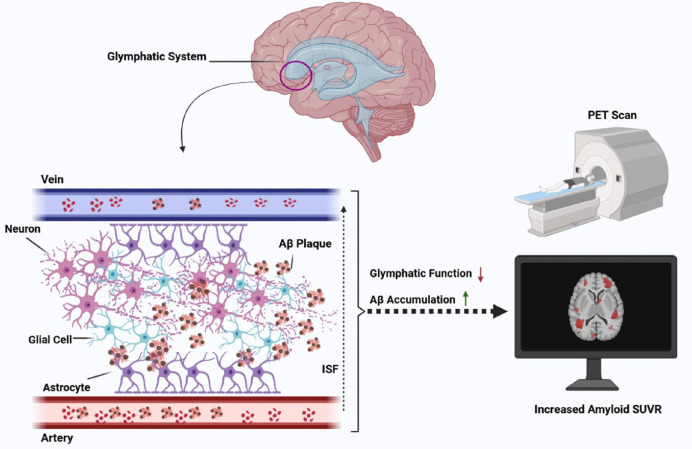
Proposed relationship between DTI-ALPS indices and amyloid accumulation in Alzheimer’s disease. Impaired perivascular fluid transport may contribute to decreased perivascular removal of Aβ and increased amyloid deposition, reflected by higher PET SUVR. Accumulation of Aβ within the ISF is proposed to interact with neurodegenerative processes and cognitive decline (Created with BioRender.com). Aβ: Amyloid-beta, PET: Positron emission tomography, SUVR: Standardized uptake value ratio, ISF: Interstitial fluid.

The results of the present study demonstrated a significant association between amyloid SUVR in temporal pole cortex, inferior temporal cortex, entorhinal cortex, and parahippocampal gyrus, and the DTI-ALPS index, particularly among patients with MCI. To observe amyloid PET in patients with AD spectrum, several compounds, such as Pittsburgh compound B (11C-PiB), 18 F-flutemetamol (FMM), and 18 F-florbetapir (FBP), are used as tracers [72]. Our study utilized FBP as the PET tracer, which has been previously shown to have a longer half-life and affinity to Aβ deposits, compared to other PET tracers [73]. Recent studies have indicated a significant association between amyloid-PET and postmortem amyloid burden among patients with AD continuum, suggesting amyloid-PET as a potential imaging method in the assessment of AD pathology [74]. Amyloid PET SUVR is typically calculated by dividing the tracer uptake in targeted cortical regions by that in a reference region, such as the cerebellar cortex or whole cerebellum, to normalize for nonspecific binding and intersubject variability [75]. This quantitative measure is reported to be a reliable indicator of Aβ deposition and is widely used to assess disease burden and progression in cognitive impairment [74]. Moreover, Aβ SUVR is derived by normalizing Aβ concentrations to a reference measure, offering a standardized index of amyloid burden that complements PET-based quantification [76]. Reduced DTI-ALPS index, reflecting altered perivascular diffusivity patterns, is associated with more aggregated amyloid deposits, as indicated by a higher amyloid SUVR, suggesting that impaired perivascular clearance can contribute to greater accumulation of amyloid deposits in both the CSF and the brain tissue [62]. In other words, amyloid burden and neurodegeneration may represent pathological processes associated with alterations in DTI-ALPS indices and cognitive decline. These findings suggest that integrating DTI-ALPS indices with amyloid PET and neurodegenerative biomarkers may help characterize disease-related alterations in early AD, which needs validation in longitudinal studies (Fig. 6).

Despite providing novel insights into the relationship between glymphatic system function, tau pathology, and amyloid burden in the AD spectrum, this study has several limitations. First, the cross-sectional design precludes causal inferences, limiting the ability to determine whether impaired glymphatic clearance precedes or follows amyloid and tau accumulation. Second, while the DTI-ALPS index provides a noninvasive proxy of the glymphatic function, it captures only perivascular water diffusivity at a single anatomical level and may not fully represent global glymphatic clearance. Additionally, plasma p-tau181 and amyloid SUVR were used as biomarkers, but longitudinal measures and simultaneous multimodal imaging could strengthen the understanding of dynamic interactions between glymphatic function and proteinopathies.

## Conclusions

In summary, this study demonstrates that reduced DTI-ALPS indices, reflecting impaired perivascular fluid transport within the glymphatic system, are associated with increased temporolimbic amyloid deposition among individuals with cognitive impairment. These findings support the concept that impaired perivascular fluid transport may contribute to region-specific vulnerability to amyloid accumulation during the early phases of AD, consistent with emerging models of neurovascular unit and barrier dysfunction in aging. Future studies should employ longitudinal designs with repeated DTI-ALPS and PET assessments to elucidate temporal relationships and further explore the mechanistic links between perivascular transport alterations and neurodegenerative processes.

## Supplementary Material

Supplementary Files

This is a list of supplementary files associated with this preprint. Click to download.

- GraphicalAbstract.tiff

## Figures and Tables

**Table 1.** Demographic characteristics.

	HC (n = 174)	MCI (n = 193)	AD (n = 43)	P value
**Age (years)**	72.26 ± 5.94	71.09 ± 7.37	74.18 ± 8.50	**0.022**
**Sex (F/M)**	79/95	111/82	27/16	**0.026**
**Education (years)**	16.57 ± 2.59	16.06 ± 2.64	15.42 ± 2.97	**0.022**
**MMSE**	28.98 ± 1.26	28.05 ± 1.69	23.42 ± 1.86	**<0.001**
**ADAS11**	5.47 ± 2.90	8.92 ± 4.49	20.09 ± 7.44	**<0.001**
**APOE 4**				**<0.001**
**Without 4**	123	100	14	
**One 4**	48	77	23	
**Two 4**	3	19	6	
**Left DTI-ALPS**	1.22 ± 0.19	1.22 ± 0.21	1.21 ± 0.26	0.945
**Right DTI-ALPS**	1.24 ± 0.18	1.21 ± 0.20	1.20 ± 0.23	0.268
**Mean DTI-ALPS**	1.23 ± 0.17	1.22 ± 0.20	1.20 ± 0.23	0.644
**Plasma p-tau181**	15.16 ± 9.98	17.77 ± 9.71	24.47 ± 10.33	**<0.001**
**Global Cortical Amyloid SUVR**	1.15 ± 0.16	1.27 ± 0.22	1.51 ± 0.21	**<0.001**
**Temporal Pole Amyloid SUVR**	0.92 ± 0.13	0.96 ± 0.16	1.07 ± 0.21	**<0.001**
**Inferior Temporal Amyloid SUVR**	1.15 ± 0.14	1.21 ± 0.17	1.39 ± 0.18	**<0.001**
**Middle Temporal Amyloid SUVR**	1.06 ± 0.15	1.13 ± 0.18	1.30 ± 0.18	**<0.001**
**Entorhinal Cortex Amyloid SUVR**	0.94 ± 0.09	0.95 ± 0.12	0.97 ± 0.13	**0.378**
**Parahippocampal Gyrus Amyloid SUVR**	0.99 ± 0.11	1.03 ± 0.13	1.14 ± 0.14	**<0.001**

Values are showed as mean ± SD or raw numbers of patients.

Results of ANOVA analysis between groups noted as p value.

APOE 4: Apolipoprotein E 4 genotype, MMSE: Mini Mental State Examination, AD: Alzheimer’s disease, ADAS 11: Alzheimer’s Disease Assessment Scale-Cognitive Subscale 11 Items, HC: Healthy Controls, DTI-ALPS: Diffusion Tensor Imaging Along Perivascular Space, MCI: Mild Cognitive Impairment, SUVR: Standardized Uptake Value, p-tau181: Tau Protein Phosphorylated at Threonine 181.

**Table 2.** Analyses Results for linear regression of DTI-ALPS indices and plasma p-Tau181 and amyloid-PET SUVR.

		Plasma p-Tau181			Global Cortical Amyloid SUVR			Temporal Pole Amyloid SUVR			Inferior Temporal Amyloid SUVR			Middle Temporal Amyloid SUVR			Entorhinal Cortex Amyloid SUVR			Parahippocampal Gyrus Amyloid SUVR			
		Beta	P-value	CI 95%	Beta	P-value	CI 95%	Beta	P-value	CI 95%	Beta	P-value	CI 95%	Beta	P-value	CI 95%	Beta	P-value	CI 95%	Beta	P-value	CI 95%	
**HC**	**Mean DTI-ALPS**	−0.073	0.314	−0.217 to 0.070	−0.013	0.856	−0.160 to 0.133	0.091	0.223	−0.056 to 0.237	−0.026	0.729	−0.173 to 0.121	0.096	0.206	−0.053 to 0.246	−0.043	0.554	−0.188 to 0.101	−0.023	0.757	−0.173 to 0.126	
	**Right DTI-ALPS**	−0.073	0.311	−0.216 to 0.069	0.016	0.833	−0.130 to 0.161	0.134	0.070	−0.011 to 0.278	−0.020	0.784	−0.167 to 0.126	0.095	0.208	−0.054 to 0.244	−0.029	0.686	−0.173 to 0.114	−0.025	0.742	−0.173 to 0.123	
	**Left DTI-ALPS**	−0.064	0.391	−0.211 to 0.083	−0.039	0.605	−0.189 to 0.111	0.038	0.621	−0.113 to 0.188	−0.028	0.716	−0.179 to 0.123	0.085	0.276	−0.069 to 0.239	−0.051	0.497	−0.199 to 0.097	−0.019	0.806	−0.172 to 0.134	
**MCI**	**Mean DTI-ALPS**	−0.083	0.277	−0.233 to 0.067	**−0.163**	**0.047**	**−0.325 to −0.002**	**−0.199**	**0.007** *	**−0.343 to −0.054**	−0.141	0.088	−0.303 to 0.021	−0.075	0.364	−0.239 to 0.088	**−0.224**	**0.002** *	**−0.363 to −0.084**	**−0.281**	**<0.001** *	**−0.428 to −0.134**	
	**Right DTI-ALPS**	−0.111	0.149	−0.261 to 0.040	**−0.202**	**0.014** *	**−0.364 to −0.041**	**−0.196**	**0.009** *	**−0.342 to −0.050**	**−0.169**	**0.041**	**−0.332 to −0.007**	−0.097	0.247	−0.261 to 0.067	**−0.240**	**<0.001** *	**−0.380 to −0.100**	**−0.290**	**<0.001** *	**−0.438 to −0.143**	
	**Left DTI-ALPS**	−0.046	0.549	−0.197 to 0.105	−0.105	0.205	−0.268 to 0.058	**−0.175**	**0.019** *	**−0.320 to −0.029**	−0.095	0.250	−0.259 to 0.068	−0.045	0.586	−0.209 to 0.119	**−0.179**	**0.013** *	**−0.320 to −0.038**	**−0.236**	**0.002** *	**−0.384 to −0.087**	
**AD**	**Mean DTI-ALPS**	−0.204	0.255	−0.561 to 0.153	−0.105	0.508	−0.425 to 0.214	−0.101	0.527	−0.423 to 0.220	−0.098	0.538	−0.420 to 0.223	−0.015	0.925	−0.346 to 0.315	−0.024	0.887	−0.359 to 0.311	−0.021	0.900	−0.356 to 0.315	
	**Right DTI-ALPS**	−0.156	0.391	−0.520 to 0.208	−0.103	0.523	−0.427 to 0.221	−0.046	0.778	−0.373 to 0.281	−0.062	0.704	−0.388 to 0.264	−0.035	0.832	−0.369 to 0.299	0.046	0.784	−0.293 to 0.385	0.047	0.781	−0.292 to 0.386	
	**Left DTI-ALPS**	−0.227	0.201	−0.581 to 0.126	−0.098	0.536	−0.416 to 0.220	−0.141	0.376	−0.459 to 0.177	−0.122	0.443	−0.440 to 0.196	0.003	0.985	−0.325 to 0.332	−0.082	0.619	−0.414 to 0.250	−0.078	0.636	−0.411 to 0.254	

* Survived FDR correction (q < 0.05).

Each cell shows the standardized Beta coefficient/p-value, extracted from the linear regression analysis between right, left, and total DTI-ALPS indices and plasma p-Tau18 and amyloid SUVR. All results were adjusted for APOE genotype, age, sex, and years of education. ALPS: Along Perivascular Space, SUVR: Standardized Uptake Value, p-tau181: Tau Protein Phosphorylated at Threonine 181, AD: Alzheimer’s disease, HC: Healthy Controls, MCI: Mild Cognitive Impairment

## Data Availability

Data used in this study are available from the Alzheimer’s Disease Neuroimaging Initiative (ADNI) database (adni.loni.usc.edu) upon application and approval.

## Abbreviations

1C-PiB: Carbon-11 Pittsburgh Compound B
Aβ: Amyloid-beta
AD: Alzheimer’s disease
ADAS: Alzheimer’s Disease Assessment Scale
ADNI: Alzheimer’s Disease Neuroimaging Initiative
AQP-4: Aquaporin-4
CSF: Cerebrospinal fluid
DTI: Diffusion tensor imaging
DTI-ALPS index: Diffusion Tensor Image Analysis along the Perivascular Space index
DWI: Diffusion-weighted imaging
FBP: Florbetapir
FMM: Flutemetamol
FSL: FMRIB Software Library
ISF: Interstitial fluid
MCI: Mild cognitive impairment
MMSE: Mini-Mental State Examination
MRI: Magnetic resonance imaging
PD: Parkinson’s disease
PET: Positron emission tomography
p-tau: Phosphorylated tau
PVS: Perivascular space
ROI: Region of interest
SCR: Superior corona radiata
SLF: Superior longitudinal fasciculus
SPECT: Single-photon emission computed tomography
SUVR: Standardized uptake value ratio
